# The Poverty-Related Stress Scale: Development and Validation of a Multidimensional Measure Assessing Poverty-Related Stressors

**DOI:** 10.1155/2023/6659030

**Published:** 2023-11-10

**Authors:** Brianna Allen, Jeffrey Klibert, Llewellyn E. van Zyl

**Affiliations:** ^1^Department of Psychology, Georgia Southern University, USA; ^2^Human Performance Management, University of Eindhoven, Netherlands; ^3^Optentia Research Unit, North-West University (Vaal Triangle Campus), South Africa; ^4^Department of Human Resource Management, University of Twente, Netherlands; ^5^Department of Social Psychology, Goethe University, Frankfurt am Main, Germany

## Abstract

**Background:**

Poverty-related stress plays a pivotal role in mediating the impact of poverty on behavioral health outcomes. However, existing research on adult poverty-related stress suffers from limited scope and inadequate measurement approaches. To address these shortcomings, our study undertakes a comprehensive investigation to develop and validate a multidimensional Poverty-Related Stress Scale (PRSS).

**Methods:**

A multistudy research design was employed to develop and validate the PRSS. Study 1 (*N* = 206) established a multidimensional framework for poverty-related stress by exploring the factor structure and internal consistency of our measure. Study 2 (*N* = 400) evaluated nuanced psychometric properties, including factorial validity, internal consistency, and temporal invariance, using confirmatory factor analyses (CFA) and modern exploratory structural equation models (ESEM). Lastly, Study 3 (*N* = 470/219) examined the criterion validity of our scale by investigating its concurrent and predictive relationships with depression, anxiety, and flourishing.

**Results:**

The findings consistently supported a hierarchal ESEM model for overall poverty-related stress, reflecting the dynamic interaction among three stressors: noise disturbance, housing dysfunction, and financial distress. This model exhibited temporal invariance, with different studies reliably measuring distinct components of poverty-related stress. Concurrent validity was demonstrated by significant associations between overall poverty-related stress and theoretically relevant constructs, such as depression, anxiety, and flourishing, at different time points. Additionally, predictive validity was established, showing poverty-related stress measured at time 1 accounted for variations in depression, anxiety, and flourishing at time 2. The results provide robust evidence for the validity and reliability of the PRSS as a tool for measuring poverty-related stress and its underlying factors.

**Conclusions:**

Our findings offer compelling preliminary support for the utility of our measure. Further research and potential clinical applications are discussed to deepen the understanding of poverty-related stress and its implications for behavioral health outcomes.

## 1. Introduction

In the United States (US), approximately 37.9 million people reside in poverty [1]. Poverty denotes situations of deprivation, both explicitly and indirectly [2]. Scholars describe poverty from various perspectives, ranging from encompassing transient or brief circumstances, persistent and continuous states, an abstract differentiation between societal definitions of wealth, an indicator of insufficiency, and/or a condition characterized by disparities in resources at individual and community levels [3]. Notably, poverty is highlighted by significant inequalities based on socioeconomic status (SES; [4]), encompassing limited access to care, financial resources, and neighborhood advantages. Consequently, these disparities directly contribute to a broad range of psychological, neurobiological, physiological, emotional, and behavioral impairments [2, 5].

However, the extent to which poverty contributes to lower indices of health, particularly behavioral health, is somewhat skewed given the low level of sophistication and comprehensiveness by which poverty indicators are measured [6, 7]. Notably, poverty-related measures often capture ill-defined, vague, and broad constructs with little meaningful theoretical relevance, contain significant errors resulting from biased frames of reference, fail to consider individuals' subjective experience with deprivation, and are administered loosely with little consideration for validity and reliability [6]. In the US, the prevailing measures of poverty-influencing policy are financial in nature and fail to consider the physical, behavioral, or social repercussions of living in impoverished conditions [7, 8]. Considering these trends, researchers need to build a better theoretical foundation for salient poverty-related indicators through rigorous construction and scientific evaluation of measures.

According to the social causation theory [9] and the family stress model [10], stress is the primary mechanism by which poverty debilitates behavioral health. Stress is a prolonged and unmediated biological response that occurs when individuals perceive a situation as dangerous and beyond their coping abilities [11]. While individuals who live in poverty report more generalized (e.g., work difficulties, interpersonal conflicts, and injuries), chronic, and toxic stressors when compared to individuals in other SES classes [12, 13], they also experience additive stressors stemming from poverty-related circumstances, contexts, and resources, including increased exposure to violence, crowded living spaces, low access to care/resources, pollution and hazardous environmental conditions, and discriminatory social practices [14]. Wadsworth and Berger [15] refer to the accumulation of stressors associated with poverty as poverty-related stress, a concept that perpetuates and exacerbates problematic health outcomes. This suggests that poverty-related stress can be classified as a systemic type of stress experienced disproportionately by individuals in lower social class groups (e.g., low SES; [16]). Poverty-related stress is often characterized by a series of intersecting events, circumstances, and challenges (a.k.a., stressors) that are perceived as strenuous, grueling, and demoralizing [17]. While proxy measures of poverty-related stress are associated with general life stressors [18], underlying features of poverty-related stress often include financial, physical, and psychosocial challenges specific to low-income and resource-depleted communities. These challenges include, but are not limited to, unreliable transport resources, interpersonal and community violence, threats to basic needs (e.g., shelter and food), increased exposure to illness, significant noise disturbances within the home, social barriers in seeking and obtaining work, difficulties accessing basic resources (e.g., showers and laundry), premature dissolution of key social supports, and financial restrictions in obtaining environmental resources [5, 15, 19–23]. Over time, these stressors overwhelm different regulatory systems making it difficult for low-income individuals to maintain adequate levels of health, engage in purposeful, goal-oriented approaches to coping, and cultivate key cognitive-affective resources (e.g., optimism and emotion regulation; [19, 23, 24]). Without intervention, poverty-related stress tends to perpetuate intergenerational cycles, hindering upward mobility, wealth accumulation, and resource acquisition for lower-income families [25].

Research consistently supports the pervasive and detrimental effects of poverty-related stress across different stages of development. Among children and adolescents, poverty-related stress disrupts regulatory processes [26] and undermines cognitive resource development, like persistence, mastery, and perceived control in managing complex circumstances [27], causing delays and difficulties with academic achievement and career development [28]. Similarly, the effects of poverty-related stress extend to adult populations. Aspects of parenting, including parental investment, responsiveness, warmth, and coping, are negatively impacted by poverty-related stressors [29]. Relationally, poverty-based stress increases the likelihood of intimate partner violence, intensifies the health consequences of intimate partner violence, and restricts access to resources to help individuals leave violent relationships [30]. With older adult populations, poverty-based indicators (e.g., lower SES) are commonly linked to lower cognitive functioning, highlighted by an increased susceptibility to dementia-related syndromes [31, 32]. These cross-developmental effects are concerning, especially when considering that poverty is intergenerationally transmitted, which causes significant difficulties in relational and marital stability and satisfaction [25].

Although poverty-related stress is linked to a wide variety of outcomes, the frequency and severity of threats to health, safety, and economic advancement are some of the most debilitating consequences [7]. This is especially true in behavioral health literature, where researchers highlight the pernicious effects on internalizing (i.e., mood and anxiety) symptoms [5, 33]. Researchers consistently report strong, prospective, and direct links between poverty-related stress, as measured by proxy concepts (i.e., economic hardship), and internalizing symptoms among family samples with low-income resources [5, 18]. Not only do poverty-related stress indices contribute to internalizing symptoms, but they also exacerbate symptoms over time [5]. Research employing different proxy measures of poverty-related stress (i.e., financial stress) also highlights strong connections with internalized symptoms, particularly depression and depressive disorders. Notably, systemic reviews conclude that indices of financial stress are strong predictors of depressive symptoms, especially for individuals residing in lower-income communities [34]. However, systemic reviews examining the effects of different poverty-related stress indicators on anxiety symptoms produced mixed findings [35]; the results indicate that the relationship between these two concepts is conditional based upon which measure of poverty-related indicators was implemented across different studies. For instance, studies employing stress related to financial difficulties as a proxy measure generated nonsignificant associations with anxiety symptoms, whereas studies employing debt stress as a proxy measure reported significant associations with anxiety.

The pernicious effects of poverty-related stress also extend to flourishing, a state of social-psychological prosperity marked by promoting and maintaining emotional vitality, social connectivity, and general positive functioning [36]. The pertinent theory asserts that poverty-related stress places significant barriers in helping lower-income individuals obtain social capital resources needed to find meaning, pursue goals, and achieve success in daily life [37], a position supported by prevailing research. Regarding social capital, individuals who experience high levels of poverty-related stress report more disruptions in family relationships and processes [38], highlighted by diminishing perceptions of social support [2]. Other disruptions stemming from poverty-related stress directly contribute to martial, interpersonal, and interparental conflicts [39–41]. Similarly, poverty-related stress undermines attempts to engage in purposeful and goal-oriented actions [19, 23], especially in forming effective coping and resilience-building elements of flourishing [42]. Across one's lifespan, poverty-related stress interferes with the ability to identify and engage in effective coping efforts [43, 44]. Finally, poverty-related stress undermines resources that promote success, including support in completing school and advantageous work/career structures [45].

These findings speak to the potential influence of poverty-related stress on behavioral health outcomes. Yet, these findings may be undermined by concerning measurement issues associated with how poverty-related stress is conceptualized and evaluated. For instance, a large portion of studies evaluating poverty-related stress use general stress measures [19], which is problematic because such measures fail to capture the unique experience and expression of stress dysregulation among individuals residing in lower-income and dilapidated conditions. To obtain a more meaningful theoretical grounding, the field of poverty-related stress needs to expand its scope and enhance its research methodologies, especially in the context of measurement construction and evaluation. Without more complex and rigorously constructed measurement tools, the field will not be able to holistically evaluate how stress-based inequities detract from different facets (physical, financial, and psychosocial) of well-being and are appropriately considered in the development of social policies and procedures across ecological systems (microsystem, mesosystem, exosystem, and macrosystem; [46]). Psychometric research must address the limitations of current poverty-related stress measures (see discussion below), identify multiple, culturally contextualized expressions of poverty-related stress, and consider the dynamic intersection of these expressions to generate greater theoretical grounding (in the form of a holistic model) for the field.

Proxy measures used to assess poverty-related stress, like economic hardship, rely on assessments that have not gone through rigorous psychometric evaluation. For instance, the Economic Hardship Questionnaire (EHQ; [47]) is one of the most used measures for poverty-related stress. Despite its popularity, there are no known studies offering a focused and comprehensive investigation regarding the EHQ's properties, calling into question its suitability to serve in this role. In the original article [47], there is little information regarding how items were developed, evaluated, and revised before administration. Because the psychometric evaluation of the EHQ was not the major thrust of the article, there were only cursory analyses conducted pertaining to the facture structure (a series of principal factor analyses), internal consistency (Cronbach's alpha), and validity (correlations with theoretically relevant variables) of the measure. No data illustrating factor validity, factor stability, temporal invariance, longitudinal structure, longitudinal measurement invariance, and predictive validity were reported. Moreover, the data offered are questionable in nature, with the unidimensional factor accounting for low levels (34.75%) of the total variance in the model and the inclusion of items with low cross-loading scores (0.35). These data violate best practices in determining measurement quality; all extracted factors should explain at least 50% of the overall variance in the model, and retained factor loadings should be greater than 0.40 [48]. The EHQ also presented some conceptual issues regarding its ability to serve as a proxy measure for poverty-related stress. Poverty indicators, like poverty-related stress, are conceptualized as multidimensional constructs [20, 21, 40], yet the EHQ only generates a unidimensional score, emphasizing financial stressors associated with living in poverty. Theorists acknowledge stress stemming from poverty conditions is more encompassing than concerns regarding money and wealth [49], often including physical (e.g., exposure to violence, crime, noise disturbance, and low community resources) and psychosocial (family turmoil, social disconnection, and parental harshness; [29, 50]) facets.

Other proxy measures for poverty-related stress also report questionable findings about measurement suitability and quality. The Multicultural Events Schedule for Adolescents (MESA; [51]) appears to cover multiple dimensions of poverty-related stress, consistent with prevailing theory. However, there are no known focused and comprehensive evaluations of the measure's psychometric properties. Like the EHQ, most of the reported metrics are offered as secondary analyses in the original article. The article does highlight metrics pertaining to factor structure, yet they seem rather incomplete. For instance, the fit confirmatory analytic model is only evaluated through two indices (*χ*^2^ and CFI), which can be problematic. Researchers largely evaluate fit from a larger pool of values because indices may not provide uniform evidence for a well-fitting model [52]. Some values are highly affected by sample size and other power metrics, which may produce misleading results if not compared against a series of diverse values. Because of this, there are some questions about the factor structure of the MESA as reported in the original article. Outside of this concern, the MESA does not appear to have gone through rigorous evaluation regarding factor stability, temporal invariance, longitudinal structure, longitudinal measurement invariance, and predictive validity, which, again, limits conclusions about its measurement quality from a poverty-related stress perspective.

It is also worth noting that most proxy measures for poverty-related stress are routinely standardized with child and adolescent populations over adult populations, which is not surprising considering poverty-related stress exacerbates internalized symptoms more for children when compared to adults [5]. However, using standardized adolescent measures of poverty-related stress with adult populations is not ideal and may contribute to a pattern of misdirection and bias regarding how adults experience and express poverty-related stress.

In combination, the current research status for poverty-related stress is still in the earlier stages of development. While movements have been made to define and conceptualize poverty-related stress, especially in behavioral health spaces, measurement quality is still a significant barrier to moving the field forward. There is a strong need to develop and rigorously evaluate new poverty-related measures instead of relying on proxy measures with unreported or questionable psychometric properties. Measures also need to be developed for lower-income adult samples, as adults are often required to navigate unique and challenging circumstances with parenting and employment when residing in impoverished environments. Moving forward, developing a new measure, validated for use with lower-income adult samples, is key to clarifying the direct, prospective, and causal relationships between poverty-related stress indicators and behavioral health outcomes.

The current study presents a multitiered approach to constructing and evaluating a new, multidimensional measure for adult poverty-related stress. Consistent with modern methods of advancing theory and measurement [53], our study will use sophisticated data-driven models (cross-sectional and longitudinal) and complex statistical analyses to evaluate a wide range of psychometric criteria, including factor structure, internal consistency, longitudinal factorial validity, temporal invariance, and criterion (concurrent and predictive) validity. Consistent with recommendations, items constructed capture unique physical (e.g., hazards, noise disturbances, exposure to pollution, and structural housing problems; [29, 50]), psychosocial (e.g., exposure to relationship instability and disconnect, family conflict, and inconsistent social support; [29, 50]), and financial (e.g., decreases in income, job instability, economic hardship, and unemployment; [54, 55]) stressors commonly reported by families residing in underresourced and impoverished communities. Study 1 conceptualizes and evaluates a multidimensional framework for poverty-related stress by investigating the exploratory factor structure and internal consistency of our measure. Study 2 evaluates more nuanced psychometric properties, including factorial validity, internal consistency, and temporal invariance, for our measure through an examination of competing confirmatory factor analyses (CFA) and modern exploratory structural equation models (ESEM). Finally, Study 3 explores the criterion validity of our measure through its concurrent and predictive relationships with depression, anxiety, and flourishing.

## 2. Materials and Methods

### 2.1. Research Design

A multistudy research design was employed to develop and validate the poverty-related stress measure. Three studies were implemented. In Study 1, a cross-sectional design explored the factorial structure of the poverty-related stress measure. Using a separate cross-sectional design, Study 2 confirmed the factorial validity of the instrument. Finally, Study 3 explored the longitudinal factor structure of the instrument and established its concurrent and predictive validity through its relationship with depression, anxiety, and flourishing, using a two-wave design.

### 2.2. Research Procedure

Institutional Review Board approval was obtained before data collection. Across all three studies, data were collected through Amazon's MTurk. Only individuals aged 18 and above, located in the US from lower-income groups, were eligible to participate. Prospective MTurk workers voluntarily enrolled in our studies and were provided with an informed consent document outlining the purpose, potential risks, and benefits, as well as the requested information for the surveys. Participants were required to provide electronic consent before proceeding with each survey. Upon volunteering, participants were given a Qualtrics survey link. Measures were presented in a randomized order to maintain anonymity, and no identifying information was collected. Upon completing the surveys, individuals received one US dollar in compensation for their participation, were thanked for their time, and were provided with a list of free and low-cost resources to alleviate any distress from participation. Most participants completed the surveys for Studies 1 and 2 within 15 minutes, while Study 3 took under 20 minutes to complete.

Based on Buchanan and Scofield's [56] guidelines to detect and manage low-quality data, response validity and data quality were assessed through a post hoc analysis of response patterns, response consistency, and completion time. First, we calculated the median response time for questionnaire completion using page timing and identified outliers, encompassing both unusually short and excessively long response times [56]. To further address response quality concerns, we adhered to Downs et al.'s [57] recommendation of utilizing the 90th percentile threshold for survey completion time as a valid criterion for exclusion. Specifically, responses that exceeded two standard deviations above/below the median response time were excluded. Additionally, we recorded the click count and click-through rates to gauge participant engagement and attentiveness during the study. Second, we proceeded to analyze the data distribution for each item and participant to identify any signs of random responses. In accordance with Buchanan and Scofield's [56] guidelines, we estimated and assessed the skewness and kurtosis of each item, aiming to detect potential deviations from uniformity. *Z*-scores for skewness and kurtosis were calculated, and an ANOVA was conducted to explore differences in these values. This distribution comparison was used to detect any indication of random responses [56]. Finally, a sensitivity analysis was performed to verify our findings' robustness. According to Buchanan and Scofield [56], if participants violated two or more of the criteria (completed questionnaires, randomness in responses, exceeding median response times for questionnaires, large deviations in answer choice distributions, and if they did not accurately complete the attention-checking questions as manipulation checks), they should be excluded from the final dataset. This rigorous approach allowed us to ensure the validity and reliability of our data for further analysis and interpretation.

### 2.3. Participants

A purposive, availability-based sampling strategy was employed to gather data for the three studies (Study 1: *N* = 206; Study 2: *N* = 400; Study 3: time 1 *N* = 470, time 2 *N* = 219). Samples were gathered from three separate groups. For Study 3, a longitudinal design was employed with a time lapse of 2 months between the first and second measures. All participants self-reported an annual household income of $25,000 or less. This criterion was established to collect data from individuals under the poverty line in the US, which is currently $27,949 for a family of four [1]. Sociodemographic information for all three studies is provided in Table 1.

In Study 1, the majority of participants identified as married (52.9%), Caucasian (60.7%) women (53.4%), aged between 31 and 40 years (36.4%), and with a college degree (35%). Similarly, in Study 2, a significant portion of participants were married (47%), Caucasian (65.8%) women (52.8%), aged between 21 and 30 years (38.5%), and possessed a college degree (35%). Lastly, in Study 3, the majority of participants were married (time 1: 56.4%; time 2: 53%), Caucasian (time 1: 74.9%; time 2: 79.5%), with a gender distribution of men at time 1 (53.6%) and women at time 2 (50.7%), aged between 31 and 40 years (time 1: 35.7%; time 2: 36.1%), and holding a college degree (time 1: 48.9%; time 2: 52.1%).

### 2.4. Measures

Participants were asked to complete different surveys across the three studies. In the first two studies, participants completed a sociodemographic form and the newly developed measure of poverty-related stress. In the third study, participants were asked to complete additional measures of depression, anxiety, and flourishing.

#### 2.4.1. Sociodemographics

A sociodemographic questionnaire was used to gather basic descriptive information about participants in all three studies. The questionnaire asked participants to self-report their age, sex assigned at birth, gender identity, race/ethnicity, marital status, and education.

#### 2.4.2. Poverty-Related Stress

**Figure 1:**
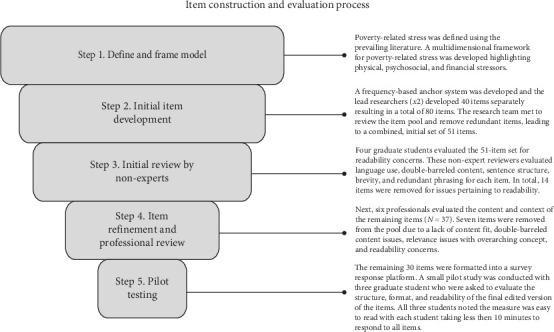
Item construction and evaluation process.

The Poverty-Related Stress Scale (PRSS) was developed to assess chronic and debilitative stress which stems from stressors associated with living in impoverished conditions. A flow chart of the screening procedures is depicted in Figure 1. The scale development process followed the International Test Commission's Guidelines [58] for scale construction and validation. A deductive approach was used to create items based on the prevailing literature on low-income barriers to well-being. As outlined by the work of Evans and Cassells [50] and Evans and Kim [29], items were developed to capture features of three poverty-related stressor categories: physical stress (e.g., substandard environmental conditions highlighted by noise, poor sanitation, and crowding), psychosocial stress (e.g., family turmoil and disconnection, low resources, and threats of harm), and financial stress (e.g., reduced income, job loss, and additional burden related to disability and illness). Initially, 51 items were generated by the research team. These 51 items captured unique contexts by which physical (*n* = 17), psychosocial (*n* = 17), and financial (*n* = 17) dimensions of poverty-related stress are expressed, including stressors pertaining to financial restrictions/hardship (e.g., “I had to make tough decisions because of lack of money”), relationship conflicts and strife (e.g., “I have felt disconnected from my family or parents because of separation or divorce”), noise disturbances within the home (e.g., “I was reluctant to go home or return home because the noise in my house was uncomfortably loud”), exposure to violence (e.g., “I encountered physical confrontations [i.e., fighting] in my home”), difficulties accessing basic needs (e.g., “I have gone hungry because there was not enough food to eat”), exposure to illness (e.g., “I was prone to sickness because of the amount of people living in my home”), dilapidated living conditions (e.g., “I have stayed in a homeless shelter, church, other public place, or another person's home because my home was not suitable to live in”), disparities in resources (e.g., “I experienced distress, because I could not locate help”), obtaining gainful employment (e.g., “I could not seem to hold a job for a long period of time”), and other environmental barriers to healthy living (e.g., “I had difficulty sleeping or doing other important things due to noise disturbances outside my home [e.g., loud neighbors, construction, neighborhood violence, public transportation, car alarms].”). A copy of the original 51 items is available upon request.

The instructions ask participants to evaluate the extent to which they experienced specific stressors over the course of the last five years. Items were assessed on a 4-point rating scale (1 = never experienced to 4 = always experienced). Consistent with best practices for establishing face- and content validity [59], the original items were reviewed by both experts and nonexperts. The initial item pool was screened by the research team and four doctoral-level students for readability, comprehension, and appropriateness for assessing the various poverty-related stressors. This team identified issues (i.e., double-barreled content, lower comprehension, and overly sophisticated language) with 14 items, which were removed from the item pool. Thereafter, six professional behavioral healthcare providers, who consistently worked with adults from underserved, underresourced, and geographically isolated areas, were recruited to review the remaining item pool (*N* = 37). These reviewers were asked to review the administration procedure, the purpose of the assessment, the clarity of the instructions, and the appropriateness of the rating scale. Further, they were requested to evaluate and rate the readability, redundancy, and content fit of the items on a 4-point rating scale (from 0 to 3) and to provide additional feedback on each item. The content validity ratio was thereafter calculated, where items receiving a score of 2 (75th percentile) were retained for the final pool. Test instructions and items were adapted based on the feedback from the reviewers. In total, 30 items (see Table 2) were retained for empirical investigation. The final pool of items was test piloted on a small group of students to evaluate administration time and comprehension of the items.

#### 2.4.3. Depression

Depression was measured using the Center for Epidemiological Studies Depression Scale (CES-D) in Study 3 [60]. The CES-D is a 20-item self-report scale designed to measure depressive symptoms experienced in the past week. The item content of the CES-D measures for depressive symptomatology is emphasized by the affective components of depressed mood [60]. All items were measured on a scale ranging from 1 (rarely or none of the time (less than 1 day)) to 4 (most or all of the time (5-7 days)). Total scores ranged from 20 to 80, with higher scores indicating greater levels of depressive symptoms. The CES-D is a reliable assessment for depressive symptoms across race, gender, and age categories [60]. The CES-D demonstrates high internal consistency, with Cronbach's alpha coefficients ranging from 0.85 to 0.90 [60]. The CES-D also demonstrates adequate test-retest stability, excellent concurrent validity by clinical and self-report criteria, and excellent construct validity [60]. In Study 3, the CES-D demonstrated excellent internal consistency (*α* = 0.96 − 0.97).

#### 2.4.4. Anxiety

Anxiety was measured using the Burns Anxiety Inventory (BAI) in Study 3 [61]. The BAI is a 33-item measure designed to assess anxious feelings, anxious thoughts, and somatic symptoms associated with generalized anxiety. Individuals indicate the extent to which anxiety symptoms have caused distress over the past week on a scale ranging from 1 (not at all) to 4 (a lot). Total scores ranged from 33 to 132, with higher scores indicating more anxiety symptoms. The BAI demonstrates high internal consistency (*α* = 0.95, [61]) and is strongly correlated with the anxiety subscale of the Symptom Checklist-90 (SCL-90), indicating high convergent validity [61]. In Study 3, the BAI demonstrated excellent internal consistency (*α* = 0.97-0.98).

#### 2.4.5. Flourishing

Flourishing was measured by the Flourishing Scale (FS) in Study 3 [36]. The FS is an 8-item scale designed to measure social-psychological prosperity. Specifically, the FS evaluates a respondent's self-perceived success in important areas like relationships, self-esteem, competence, purpose, and optimism. The FS is rated on a scale from 1 (strongly disagree) to 7 (strongly agree). Scores ranged from 8 to 56, with higher scores signifying greater levels of flourishing. In terms of psychometric properties, the FS demonstrates high internal consistency (*α* = 0.87) and temporal reliability [36]. The FS is also strongly associated with other psychological well-being scales [36]. In Study 3, the FS demonstrated excellent internal consistency (*α* = 0.94 − 0.95).

### 2.5. Statistical Analyses

Data were analyzed by both JASP 0.15 [62] and Mplus 8.8 [63]. The structural equation modeling (SEM) framework with the maximum likelihood (ML) estimation method was used to explore the instrument's factorial, concurrent, and predictive validity (To test assumptions, descriptive statistics (means, standard deviations, skewness, kurtosis, and Shapiro-Wilk's distribution tests) and Pearson's correlations were estimated and reported in Tables 3(a)–3(c). For Study 1, the mean scores were used. For Study 2 and Study 3, factor scores were computed for the H-ESEM model (latent mean of zero). The full information maximum likelihood estimation method (FIML) was used to manage missing data.

In Study 1, an exploratory factor analytical (EFA) strategy through the SEM framework was used to investigate the factorial structure of the PRSS. The factorability of the instrument was first determined through the Kaiser-Meyer-Olkin (KMO) approach and Bartlett's sphericity test. A KMO value greater than 0.60 and a significant chi-square on Bartlett's sphericity test indicate that meaningful factors could be extracted from the data [64]. Thereafter, different competing EFA models were estimated via the SEM framework and subsequently compared. An oblique rotation was used to extract factors with eigenvalues larger than 1 [65]. Competing exploratory models were estimated and compared based on conventional data-model fit indices (c.f.  Table 4) and measurement quality indicators. Measurement quality was established when items loaded significantly on their respective factors (factor loading >0.40; *p* < 0.05); all extracted factors should explain at least 50% of the overall variance in the model, and items should represent a single (not multiple) factor [48]. Items loading on multiple factors were identified and systematically removed [66]. Finally, based on the best-fitting exploratory model, the reliability of the factors was estimated through both McDonald's omega (*ω* > 0.80) and Cronbach's alpha (*α* > 0.70: [67]).

In Study 2, the factorial validity of the instrument was explored through estimating and comparing different competing measurement models. Both traditional CFA and ESEM frameworks were estimated based on the results of the previous study and systematically compared to find the best-fitting factorial model for the data. The CFA models were specified in accordance with the assumptions of the independent cluster modeling approach, where items were only permitted to represent a single factor, and no cross-loadings were permitted. To estimate the bifactor CFA model, all factors were specified as orthogonal (i.e., not permitted to co-vary) and a target rotation was used to specify an overall (general) poverty-related stress factor coupled with three specific factors. The ESEM models were specified per Van Zyl and ten Klooster's [48] best practice guidelines. ESEM is a statistical technique used to assess the factorial validity of constructs in a complex model by combining elements of both exploratory and confirmatory factor analysis [68]. In the context of poverty-related stress, ESEM offers distinct advantages over traditional Confirmatory Factor Analytical models. Poverty-related stress is multifaceted and characterized by dynamic interactions among various stressors. Unlike CFA's, ESEM's flexibility in allowing cross-loadings and residual covariances enables a more accurate representation of these dynamic relationships, aligning better with the theoretical construction of poverty-related stress. This interaction cannot be captured by traditional CFA's where cross-loadings are constrained to be zero [48]. ESEM is also better suited for multidimensional models or instruments that have complex factorial structures [48]. It can handle situations where items may load on more than one factor which more accurately captures the complexity of multidimensional constructs [68]. ESEM also provides better model fit indicators than traditional CFAs, especially when dealing with complex data or when there is some uncertainty about the extent of the interaction between underlying latent factors [48]. The inclusion of residual covariances and cross-loadings helps improve model fit and reduces potential model misspecifications and potential biases. In traditional CFAs, models suffer from misspecification bias as a result of strict loading constraints, leading to potential distortions in the results. ESEM's more flexible approach reduces this bias and improves the accuracy of parameter estimates and standard errors [48]. Similarly, by allowing cross-loadings (but constrained to be as close to zero as possible), ESEM can provide more interpretable results as it permits items to be associated with factors that are theoretically relevant and practically meaningful [68]. Further, ESEM also controls for wording effects, and differences in the experience of the different stressors [69]. ESEM thus provides a more nuanced and comprehensive understanding of the underlying factors influencing poverty-based stress, leading to more reliable and valid conclusions to be drawn about the underlying factorial structure of the instrument [68].

Within the ESEM models, the CFA models were respecified to allow for cross-loadings between items and nontarget factors. These cross-loadings were constrained to be as close to zero as possible and a target rotation was used [48]. For the bifactor ESEM model, a single general factor of overall poverty-related stress was estimated, coupled with three specific factors. Cross-loadings were only permitted between the specific factors and constrained to be as close to zero as possible. The Mplus code was generated by De Beer and Van Zyl's [70] ESEM code generator.

Both the CFA and ESEM models were then contrasted and compared based on both model fit indices (c.f.  Table 4) and measurement quality indicators. Measurement quality was evaluated by inspecting the standardized factor loadings (e.g., *λ* >0.40), the item uniqueness (e.g., >0.10, but <0.90), and the level of internal consistency of the various factors as discussed previously [71]. Additionally, the measurement quality for the CFA and ESEM bifactor models was assessed by establishing a well-defined general factor (with significant factor loadings) and relatively well-defined specific factors [72]. Models that met both the model fit and measurement quality criteria were retained for further analysis.

In Study *3*, longitudinal factor analysis (LFA) and longitudinal measurement invariance (LMI) were used to determine the instrument's factorial stability and internal consistency over time. For the LFA, associated factors from time 1 were permitted to co-vary with their respective counterparts at time 2. Factors from time 1 needed to be strongly associated with their counterparts in time 2 (*r* =0.80, *p* <0.05). All factorial models tested in Study 2 were used as input for the longitudinal factor analytic models. The estimated models were again assessed for data-model fit and measurement quality and subsequently compared. To assess the instrument's temporal equivalence, LMI was assessed for the best-fitting LFA model. Here a series of increasingly restrictive models were estimated, evaluated, and subsequently compared. Three models were estimated and compared: (a) a configural invariance model (similar factor structures over time), (b) a metric invariance model (similar factor loadings over time), and (c) a scalar invariance model (similar intercepts over time). Chen's [73] criteria were used to compare models and determine if there were significant differences between the two time points: changes in RMSEA (*Δ* <0.015; *p* <0.05), SRMR (*Δ* <0.02 for configural versus metric/scalar; *Δ* <0.01 for metric versus scalar), CFI (*Δ* <0.01), and TLI (*Δ* <0.01). Further, no statistically significant difference in chi-square between time points should be present (*p* <0.05; [66]).

Finally, the concurrent and predictive validity of the instrument was explored through estimating separate structural models. Here, the best-fitting measurement model was used as an exogenous factor and regressed on depression, anxiety, and flourishing (as endogenous factors). For concurrent validity, overall poverty-related stress at time 1 was regressed on depression, anxiety, and flourishing at time 1. Similarly, overall poverty-related stress at time 2 was also regressed on the endogenous factors at time 2. For predictive validity, overall poverty-related stress at time 1 was regressed on the endogenous factors at time 2. The time span between the first and second assessments was two months. A significant relationship between the factors was required to establish the respective types of validity (*p* < 0.01; [71]).

## 3. Results

The results of the exploratory factor analyses (Study 1), competing measurement models, item-level descriptive and internal consistency (Study 2), longitudinal factor analysis, and concurrent/predictive validity (Study 3) are tabulated, reported, and discussed separately below.

### 3.1. Study 1: Exploratory Factor Analysis

A competing EFA modeling approach was used to determine the factor structure of the PRSS. First, the factorability of the instrument was established through the KMO measure (KMO = 0.93) and Barlett's sphericity assessment (*χ*^2^ = 193.276; *p* < 0.01). Given these findings, meaningful factors were extracted. Second, an initial EFA was conducted, and the communalities were inspected. Fifteen items with communalities lower than 0.40 were removed ([71]: c.f. Table 2). The final set of fifteen items was then used as input for further analysis.

Third, a series of six EFA models were then estimated. Only three of these models converged: (a) Model 1—a unidimensional model (*χ*^2^_(206)_ = 439.09; df = 90; *χ*^2^/df = 4.88; CFI = 0.94; TLI = 0.79; RMSEA = 0.14 [0.126, 0.150], *p* < 0.01; SRMR = 0.08; AIC = 7120.45; BIC = 7270.20; eigenvalue factor 1 = 7.75; *R*^2^ = 51.69%); (b) Model 2—a two first order factorial model (*χ*^2^_(206)_ = 242.598; df = 76; *χ*^2^/df = 3.19; CFI = 0.91; TLI = 0.88; RMSEA = 0.10 [0.089, 0.118], *p* < 0.01; SRMR = 0.05; AIC = 6951.95; BIC = 7148.30; eigenvalue factor 1 = 7.75; *R*^2^ = 51.69%; eigenvalue factor 2 = 1.57; *R*^2^ = 10.46%), and (c) Model 3—a three first order factorial model (*χ*^2^_(206)_ = 107.87; df = 63; $\chi^{2}/\underset{¯}{\text{df}}=1.71$; CFI = 0.98; TLI = 0.96; RMSEA = 0.06 [0.039, 0.077], *p* > 0.01; SRMR = 0.02; AIC = 6951.95; BIC = 7148.30; eigenvalue factor *1 = 7.75*; *R*^2^ = 51.69%; eigenvalue factor 2 = 1.57; *R*^2^ = 10.46%; eigenvalue factor 3 = 1.10; *R*^2^ = 7.27%). Only Model 3 met the measurement quality criteria.

The item-level parameter estimates of this model are presented in Table 5. Three factors could therefore be extracted from the data. Table 5 indicates that all factor loadings exceeded the 0.40 threshold criteria, and each factor produced acceptable levels of internal consistency (McDonald's omega > 0.80 and Cronbach's alpha > 0.70). Cumulatively, all three factors explained more than 50% of the total variance in the model. The three factors were labeled noise disturbance, housing dysfunction, and financial distress based on the thematic overlap in item content. The Geomin factorial correlation showed that noise disturbance was positively related to housing dysfunction (*r* = 0.73; *p* < 0.01) and financial distress (*r* = 0.41; *p* < 0.01). Further, housing dysfunction was also positively related to financial distress (*r* = 0.43; *p* < 0.01). The findings suggest that the three factors are distinct and explain the unique variance in the overall model.

### 3.2. Study 2: Competing Measurement Models and Item Level Parameter Estimates

To further investigate the factorial validity of the PRSS, a competing confirmatory factor analytical strategy was employed. Seven theoretically informed competing measurement models drawing from both the CFA approach and the ESEM framework were estimated and compared. Measured items were used as indicators for first-order latent factors; no items were removed, and error terms were not permitted to covary.

The following seven models were estimated and compared:

- Model 1: a unidimensional first-order CFA model was specified, where all 15 items were loaded directly onto an overall poverty-related stress factor
- Model 2: a correlated three first-order CFA model was estimated, where five items were loaded onto the noise disturbance factor, six items on the housing dysfunction factor, and four items on the financial distress factor
- Model 3: a second-order CFA model was specified based on the indicators in Model 2. The three first-order factors were specified to load onto a higher-order poverty-related stress factor
- Model 4: a bifactor model with one general poverty-related stress factor and three specific factors (noise disturbance, housing dysfunction, and financial distress) was estimated. These factors were specified as orthogonal
- Model 5: an ESEM model with correlated first-order factors was specified based on Model 2. In this model, all items were specified to load onto a priory factor; however, cross-loadings between factors were permitted but targeted to be as close to zero as possible
- Model 6: a hierarchal ESEM model with one higher-order poverty-related stress factor, comprised of three first-order factors (noise disturbance, housing dysfunction, and financial distress), was specified. Like Model 5, cross-loadings were permitted but targeted to be close to zero
- Model 7: a bifactor ESEM model was estimated with one general poverty-related stress factor and three specific factors (noise disturbance, housing dysfunction, and financial distress). All items are loaded directly onto the general factor. Items were then targeted to load onto the a priori first-order factorial models. Factors were orthogonal, and cross-loadings between specific factors were allowed and constrained to be as close to zero as possible

Table 6 summarizes the model fit statistics for each of the seven competing measurement models. Upon initial inspection, the results showed that the bifactor CFA (Model 4) and bifactor ESEM models (Model 7) demonstrated excellent data-model fit. However, both models failed the measurement quality criteria, where target items did not load significantly onto their *a priori* factorial models, and two of the three specific factors were poorly defined. Factor loadings also did not exceed the minimum thresholds (*λ* > 0.40; *p* < 0.01). Therefore, both the bifactor CFA and bifactor ESEM models were rejected from further consideration.

**Figure 2:**
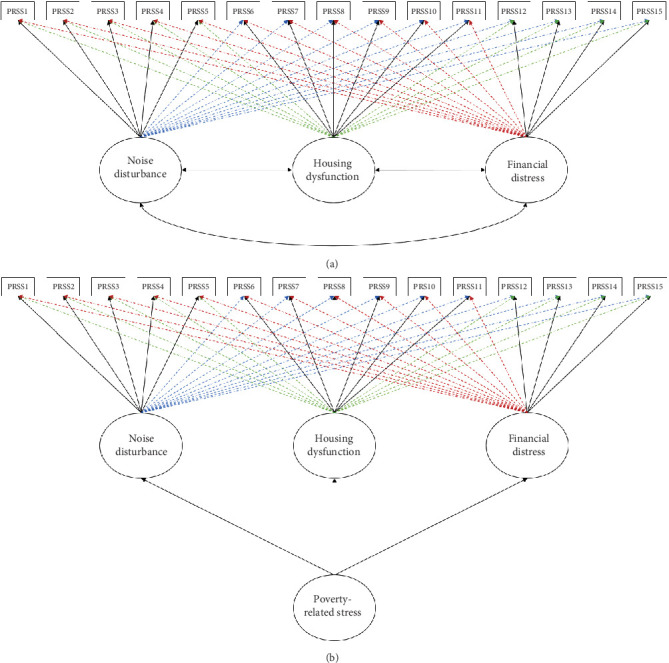
Graphical depiction of the two models that fitted the data best and demonstrated excellent measurement quality in study 2. (a) Model 5: correlated three first-order ESEM model. (b) Model 6: hierarchal ESEM model.

Moving forward, the results showed that the less restrictive three first-order factorial ESEM (Model 5) and its hierarchal ESEM counterpart (Model 6) fitted the data best (*χ*^2^_(400)_ = 86.05, *p* > 0.01; df = 63; CFI = 0.99; TLI = 0.99; RMSEA = 0.03 [0.010, 0.045], *p* > 0.01; SRMR = 0.02; AIC = 13476.434; BIC = 13763.82), demonstrating excellent measurement quality (c.f.  Table 7; Figures 2(a) and 2(b)). The results support the presence of an overall poverty-related stress factor, which is a function of a dynamic interaction among three factors: noise disturbance, housing dysfunction, and financial distress. Models 5 and 6 revealed acceptable standardized factor loadings (*λ* > 0.40; *p* < 0.01), low standard errors, acceptable levels of item uniqueness (*δ* < 0.10 but >0.90; *p* < 0.01; [71, 74]), and acceptable levels of internal consistency (McDonald's omega > 0.80 and Cronbach's alpha > 0.70; [71]). As a result, the higher-order ESEM model, which is comprised of a higher-order “poverty-related stress” factor deconstructed through a dynamic interaction between three first-order factors (noise disturbance, housing dysfunction, and financial distress), was retained for further analysis (The higher-order ESEM model (Model 6) was retained for further analysis given that no distinction between model fit can be made at a first-order factorial level (Model 5). The higher-order factorial model is also more in line with our theoretical assumptions).

### 3.3. Study 3.1: Longitudinal Factor Analysis and Longitudinal Measurement Invariance

Study 3 focused on determining the factorial stability of the PRSS over time using longitudinal confirmatory factor analysis (L-CFA). For transparency, L-CFAs were conducted on each of the models estimated in the previous study. In each L-CFA model, the measurement model identified in time 1 was specified to covary with its *a priori* measurement model counterpart in time 2.

**Figure 3:**
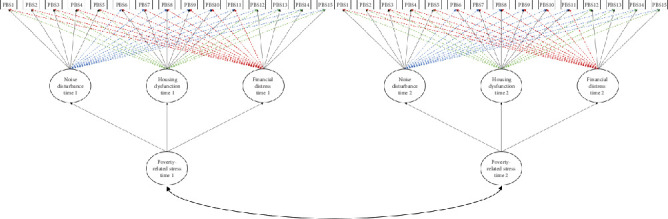
Graphical depiction of the hierarchical longitudinal ESEM (Model 6) in evaluating the overall poverty-related factors scores across time.

The results, summarized in Table 8, indicated that only 6 out of the 7 models could converge. The results further showed less restrictive longitudinal ESEM models again fitted the data best. Model 6, however, showed to fit the data slightly better than Model 5 (Δ *χ*^2^_(470)_ = −35.07; Δdf = 2; ΔAIC = −0.32; ΔBIC = −47.37). Further, the overall poverty-related stress factor at time 1 was strongly associated with the overall poverty-related stress factor at time 2 (*r* = 0.982; S.E. = 0.01; *t* = 73.68; *p* < 0.05; compare Figure 3). The hierarchical longitudinal ESEM Model 6 was therefore retained for the LMI estimation.

Next, the scale's temporal equivalence was assessed through LMI. The results, summarized in Table 9, showed no statistically significant differences in chi-square (*p* < 0.05), RMSEA (Δ < 0.015; *p* < 0.05), SRMR (Δ < 0.015), CFI (<0.01), and TLI (<0.01) analytics between the configural, metric, and scalar invariance models [66]. Taken together, the hierarchal ESEM model 6 demonstrated consistency over time. This means that the scale demonstrated temporal equivalence, indicating that the instrument's factorial structure, factor loadings, and intercepts remained consistent across time, making it suitable for concurrent and predictive validity estimations. This model is therefore retained for further analysis.

### 3.4. Study 3.2: Concurrent and Predictive Validity

To estimate the concurrent and predictive validity of the PRSS, three separate structural models were estimated with the hierarchical ESEM factorial model. The higher-order poverty-related stress factor was specified as an exogenous factor, and depression, anxiety, and flourishing as endogenous factors. In all three models, depression and flourishing were estimated as unidimensional models and anxiety as a higher-order factorial model comprised of three first-order factors. Items on depression were specified as categorical, and the WLSMV estimator in Mplus was subsequently used. The results of both the concurrent and predictive validity estimates are summarized in Table 10.

Descriptive statistics and correlations among the study's main variables are depicted in Tables 3 (a), 3(c). For concurrent validity, poverty-related stress at time 1 was regressed on depression, anxiety, and flourishing at time 1. This model adequately fitted the data: (*χ*^2^_(470)_ = 4855.58; df = 2732; CFI = 0.90; TLI = 0.90; RMSEA = 0.04 [0.039, 0.043], *p* > 0.01; SRMR = 0.05). Poverty-related stress at time 1 was directly and positively associated with depression (*β* = 0.85; S.E. = 0.02; *p* < 0.01; *R*^2^ = 0.71) and anxiety (*β* = 0.94; S.E. = 0.01; *p* < 0.01; *R*^2^ = 0.88). As anticipated, poverty-related stress at time 1 was directly and negatively associated with flourishing at time 1 (*β* = −0.15; S.E. = 0.04; *p* < 0.01; *R*^2^ = 0.02).

Similarly, poverty-related stress at time 2 was also directly and positively associated with depression (*β* = 0.68; S.E. = 0.04; *p* < 0.01; *R*^2^ = 0.46) and anxiety (*β* = 0.91; S.E. = 0.02; *p* < 0.01; *R*^2^ = 0.83) and negatively linked with flourishing at time 2 (*β* = −0.21; S.E. = 0.06; *p* < 0.01; *R*^2^ = 0.04). This model also adequately fitted the data (*χ*^2^_(219)_ = 3862.23; df = 2735; CFI = 0.90; TLI = 0.90; RMSEA = 0.04 [0.040, 0.047], *p* > 0.01; SRMR = 0.07). Thus, there is sufficient evidence to support the concurrent validity of the instrument. Regression models depicting the concurrent relationships between poverty-related stress domains and the three behavioral health indices are depicted in Table 11.

To establish predictive validity, poverty-related stress at time 1 was regressed on depression, anxiety, and flourishing at time 2. This model adequately fitted the data: (*χ*^2^_(283)_ = 3845.28; df = 2739; CFI = 0.91; TLI = 0.90; RMSEA = 0.03 [0.027, 0.031], *p* > 0.01; SRMR = 0.07). Notably, poverty-related stress at time 1 was directly and positively associated with depression (*β* = 0.58; S.E. = 0.05; *p* < 0.01; *R*^2^ = 0.33) and anxiety (*β* = 0.74; S.E. = 0.03; *p* < 0.01; *R*^2^ = 0.55) at time 2. Poverty-related stress at time 1 was also directly and negatively associated with flourishing at time 2 (*β* = −0.24; S.E. = 0.06; *p* < 0.01; *R*^2^ = 0.06). In response to these findings, there is sufficient evidence to support the predictive validity of the instrument. Regression models depicting the predictive relationships between poverty-related stress domains and the three behavioral health indices are depicted in Table 11.

The results of the study demonstrate that the PRSS is a valid tool for measuring stress related to poverty. Results highlight significant relationships between poverty-related stress and depression, anxiety, and flourishing at both time 1 and time 2, indicating good concurrent validity. Additionally, poverty-related stress at time 1 predicts depression, anxiety, and flourishing at time 2, providing evidence for predictive validity.

## 4. Discussion

The study conceptualized, developed, and validated a multidimensional measure of poverty-related stress. Specifically, it explored (Study 1) and confirmed the factorial structure of the PRSS and examined its internal consistency (Study 2). Further, the study examined the longitudinal factorial validity, temporal invariance, and criterion (concurrent and predictive) validity of the PRSS through its association with depression, anxiety, and flourishing over time (Study 3). The findings consistently supported a hierarchal ESEM model for overall poverty-related stress, which is a function of a dynamic interaction among three stressors: noise disturbance, housing dysfunction, and financial distress. This model was invariant over time, and different components of stress-related poverty were reliably measured in different studies. Concurrent validity was established by demonstrating significant associations between overall poverty-related stress and theoretically relevant constructs such as depression, anxiety, and flourishing at different time points. Further, predictive validity was established by showing that poverty-related stress measured at time 1 accounted for variations in depression, anxiety, and flourishing at time 2. The results provide strong evidence for the validity and reliability of the PRSS as a tool for measuring poverty-related stress and its underlying factors.

### 4.1. The Poverty-Related Stress Framework

The first objective of this study was to conceptualize, develop, and evaluate a multidimensional framework for poverty-related stress. In Study 1, we aimed to develop and evaluate a new measure for poverty-related stress and explore its factorial structure within a small sample of lower-income adults residing in the US. Our empirical findings support the notion that “poverty-related stress” is a state of prolonged physical, emotional, or psychological strain arising from living in unsafe conditions or experiencing severe financial hardship brought on by a dynamic interaction among three stressors: (a) noise disturbance, (b) home dysfunction, and (c) financial distress. Noise disturbance refers to feelings of stress or fatigue brought on by the prolonged presence of loud or disruptive sounds within or outside an individual's home. This stressor causes difficulties sleeping or completing important tasks, resulting in a reluctance to return or a desire to leave home when the noise becomes overbearing. Home dysfunction refers to a variety of stressors related to dilapidated living conditions and inadequate housing resources, including food insecurity, social disconnection from family and friends, and threats to maintaining available environmental resources. Finally, financial distress refers to stress or strain directly associated with financial hardship highlighted by difficult decisions/sacrifices, worry over obtaining basic needs, and apprehension concerning sudden or unexpected changes in living circumstances.

These results are broadly in line with the prevailing poverty-related literature, which emphasizes that stress stemming from poverty is attributable to the interaction between internal (e.g., financial distress) and external stressors (e.g., noise pollution, crowding, and violence in the community; [10, 49]). We believe this interaction is well-considered in the conceptualization and validation of our measure. Notably, results highlight that the overarching construct of poverty-related stress is best conceptualized through a dynamic interaction between internal sources (i.e., perceptions of worry, uncertainty, and fear) and external pressures (i.e., low access to community resources, the presence of physical hazards, and threats from systems and institutions). Further, item content within the PRSS captures variation in chronic environmental structures (i.e., neighborhood violence, physical hazards, and unreliable resources). All of these features are adequately captured through the items retained in the final measurement model and considered in each facet of poverty-related stressors.

Consistent with the overarching literature [29, 50, 55, 75], we expected results to furnish three dimensions of poverty-related stress, with strong content links to physical, psychosocial, and financial sources of risk. The results did produce three first-order factors. However, the content of these factors did not fit neatly into the expected tripartite framework. Although content associated with financial risk was clearly outlined as a unique factor within our model, physical and psychosocial risk elements were not clearly delineated as separate dimensions. Instead, our results identified two factors with seemingly different physical risk themes, noise disturbance and home dysfunction, and no clear-cut psychosocial factor. Instead, psychosocial factors such as home dynamics and family relationships form part of, but are not clearly separate from, the three different stressors. Content associated with condemnation, poor maintenance, eviction, low resources, and dependence upon shelters was expected to load onto a general home dysfunction theme, as well as content associated with noise difficulties. However, it appears that difficulties with noise constitute a unique set of stressors from other general physical risk stress within the home. This separation may be explained by biopsychosocial models, whereby stressful environmental stimuli (e.g., noise) in low-income homes uniquely contribute to added levels of sleep deprivation [76] and mental health problems [77]. Given these patterns, it appears that noise disturbance constitutes a distinct physical or environmental element of poverty-related stress. Additionally, the lack of a definitive and observable psychosocial factor was surprising, as research consistently highlights the importance of psychological and social processes in the formation of poverty-related stress [20, 22]. In a review of item content, psychosocial elements are present yet interspersed across all three first-order factors. This pattern suggests that psychosocial manifestations of poverty-related stress are highly saturated in physical (noise and home dysfunction) and financial dimensions.

### 4.2. The Psychometric Properties of the PRSS

The second objective of the paper was to evaluate the factorial validity, internal consistency, and temporal invariance of the PRSS. In Study 2, a series of restrictive independent cluster modeling CFA and less restrictive ESEM models were estimated and subsequently compared. When considering factorial validity, the results showed all CFA and ESEM models fitted the data with the exclusion of the unidimensional CFA model, indicating significant evidence for the multidimensionality of poverty-related stress. Upon further inspection, the results revealed neither the CFA nor the ESEM bifactor models met the measurement quality criteria, suggesting poverty-related stress is a function of an interaction among different stressors and not an overall experience separate from, yet related to, the three stressors.

Further, the three first-order factor ESEM (comprised of noise disturbance, home dysfunction, and financial distress) and hierarchal ESEM model with an overall poverty-related stress factor and three first-order factors fitted the data significantly better than their CFA counterparts. This implies that there is a dynamic interaction among noise disturbance, home dysfunction, and financial distress which results in an overall experience of poverty-related stress. Poverty-related stress is not merely a function of the sum of different stressors but rather the outcome of an interaction among different stressors. In other words, financial distress may, for example, influence experiences of home dysfunction and noise disturbances as financial hardships make it difficult to afford sufficient accommodation resources to meet basic needs. This interaction among factors cannot adequately be modeled through traditional CFA approaches, where it is assumed each factor functions in isolation from one another [69]. When allowing for cross-loadings between items and factors (constrained to be close to zero), the ESEM approach presents a more accurate representation of how poverty-related stress occurs or is experienced in real-world terms [68, 69].

These assumptions were further supported by the longitudinal factor analysis conducted in Study 3. When considering the factorial stability of the PRSS over time, our results suggest only two ESEM models (the first-order factor model and the hierarchal ESEM model) fit the data and meet the measurement quality criteria. This implies that these models produced similar factorial structures at both time points. To further explore factorial equivalence over time, longitudinal measurement invariance was estimated based on the hierarchal ESEM model. The results supported the configural, metric, and scalar invariance of the PRSS's hierarchal ESEM model, indicating the PRSS measures overall poverty-related stress equally and consistently across time, and similar factor structures, factor loadings, intercepts, and error variances were found at both time points. Taken together, our results support the PRSS's temporal stability, which implies that latent mean differences can be estimated and used to track changes in poverty-related stress over time. Further, the results imply that latent mean changes represent actual temporal changes in the factors over time and not changes in the meaning of the constructs [66, 78]. Therefore, meaningful comparisons across time can be made with the instrument [78].

The results also demonstrated that PRSS reliably measures different components at the lower- and upper-bound limits, with Cronbach's alphas and McDonald's omegas exceeding the suggested thresholds (*α* > 0.70; *ω* > 0.70; [67]). These results imply that the hierarchal ESEM model of the PRSS is a valid and reliable measure to assess poverty-related stress over time.

### 4.3. Criterion Validity: Poverty-Related Stress, Depression, Anxiety, and Flourishing

The final objective of the study was to explore the criterion validity of the PRSS through its concurrent and predictive relationships with depression, anxiety, and flourishing at different time points. The results showed that concurrent and predictive validity could be established, with overall poverty-related stress being significantly related to depression, anxiety, and flourishing within and between time points.

Regarding concurrent validity, the results highlight that overall poverty-related stress was positively related to depression and anxiety at time 1 and time 2, suggesting individuals with high self-reported levels of poverty-related stressors also report higher levels of depression and anxiety-related symptoms. From a theoretical level, these findings validate anxiety models, where living in poverty and experiencing prolonged financial distress may increase debilitative physiological, affective, and cognitive features of fear and worry stemming from a perceived inability to meet basic physiological and security needs [79]. For example, an individual living in poverty who constantly worries about being able to pay rent or provide food for their families is more likely to be anxious and fearful in the future. Similarly, this preoccupation with addressing current and future needs due to financial hardship may contribute to feelings of hopelessness, helplessness, and social isolation, ultimately increasing the risk for depressive disorders [19]. Furthermore, poverty-related stress was also negatively associated with flourishing at both time points. This finding is consistent with research suggesting individuals who report high levels of poverty-related stress find it difficult to experience joy and fulfillment in everyday activities and realize life goals [80].

In terms of predictive validity, the associations between overall poverty-related stress at time 1 and depression, anxiety, and flourishing at time 2 were evaluated. Similar to the previous findings, the results revealed positive relationships between poverty-related stress and measures of psychopathology (i.e., depression and anxiety), as well as a negative relationship with flourishing over time. In total, these findings validate poverty-related stress as a risk factor [81] for different indices of mental health conditions [20].

### 4.4. Limitations and Recommendations

While this study presented a valid and reliable measure for poverty-related stress, several limitations are worth noting to inform future research. First, the samples drawn for the study are restricted in terms of ethnicity, gender identity, and geographic location. Moreover, we failed to adequately assess for sexual orientation identities within our sample, which appears significant given that LGBTQ+ individuals are disproportionately represented in US homelessness and impoverished statistics and trends [82]. These restrictions limit the generalizability of the findings. More diverse samples are required to extend the generalizability of our findings, especially to groups with intersecting identities (e.g., lower-income and rural statuses). Further, despite participants self-identifying with a lower-income identity status, there was no way to confirm whether this status accurately represented the participants' financial situation. Second, the study relied heavily on MTurk to obtain data which presents several challenges. It is quite possible that individuals living in deep poverty [83] may not have been able to participate due to a lack of internet and communication services required to complete the survey. Third, various strategies were employed to ensure quality data (e.g., attention checks and post hoc analysis of response patterns) which resulted in a significant number of participants being removed from the study due to validity concerns. It is unknown if participants removed from the data were qualitatively different from those who were retained. Future research should employ more diversity in recruitment practices and distribution methods to ensure individual differences within a low socioeconomic sample are accounted for. Fourth, for the longitudinal studies, a relatively short assessment period (i.e., two months) was established between time 1 and time 2. Although this helped establish the instrument's temporal stability, it limits the PRSS's clinical significance; the short period between assessments could not completely or comprehensively account for variations or changes in poverty-related stress, anxiety, depression, or flourishing. Future research should aim to implement a longer evaluation period, between 3 and 6 months, to adequately capture this variation. Fifth, only self-report measures were used, and no measures for social desirability were implemented. Thus, there is no way to assess the reliability of responses. Future research should aim at creating a behavioral checklist based on the PRSS to assess it through observational methods. Finally, in terms of concurrent validity, we failed to include a measure of general stress functioning, like perceptions of stress, in our evaluation. The inclusion of such a measure is important in validating whether poverty-related stress dimensions are connected to general stress processing and experience. This connection is important in defending the theoretical position that poverty-related stress is a unique and culturally contextualized form of stress. Moving forward, it will be important to extend the psychometric validation of our measure by evaluating its associations with more generalized facets of stress (e.g., life stress and stress perceptions).

### 4.5. Innovation and Future Directions

The development and psychometric evaluation of our measure offer a significant extension to the poverty-related stress literature. Notably, this is the first measure to conceptually ground poverty-related stress into a meaningful framework for lower-income adult populations; poverty-related stress is the byproduct of intersecting environmental (hazardous living conditions, noise disturbances) and financial stressors. Our ability to evaluate the conceptual nature of poverty-related stress is directly tied to our design (multitiered studies) and plan of statistical analysis, where the use of ESEM expanded the scope by which we could evaluate poverty-related stress, especially when compared to traditional CFA modeling. Our findings also highlighted multiple poverty-related dimensions, which is consistent with the prevailing literature [20, 21, 40]. In this vein, our measure provides researchers with greater depth and breadth of coverage when compared to proxy and unidimensional measures of poverty-related stress, like financial hardship. Finally, our findings present a rigorous and comprehensive evaluation of psychometric soundness, which is not often reported with commonly administered proxy measures of poverty-related stress, like the EHQ and MESA. There is firm evidence for the factor structure and stability, internal consistency, temporal invariance, and concurrent and predictive validity of PRSS as a meaningful assessment tool. Overall, the PRSS makes a significant contribution to the literature, offering a unique pathway by which the literature can move out of the early phases of conceptual grounding and evaluation into more rigorous investigations of how poverty-related stress impacts behavioral health domains in diverse populations of lower-income adults.

Moving forward, it will be important for researchers to capitalize on our findings and further evaluate the links between poverty-related stress and different mental health outcomes. Because of difficulties in randomizing (e.g., ethics) or replicating poverty-related stress in a lab setting, evaluating the causal link between poverty-related stress and mental health using more traditional experimental designs will be difficult. Instead, it may be important to use experimental designs to evaluate how the effects of poverty-related stress can be offset in minimizing depression and anxiety symptoms. For instance, constructing randomized control procedures to evaluate the effectiveness of integrated care practices (simultaneously increasing access to health resources and promoting positive coping behaviors; [20]) may clarify the conditions by which poverty-related stress exerts its effects on depression and anxiety in low-income communities. Such investigations also hold promise in elucidating the parameters by which clinicians can employ effective prevention and intervention programs.

It is also essential for researchers to investigate other forms of validity (e.g., incremental validity) in evaluating the psychometric properties of the PRSS. Other culturally responsive models of strain [84] conceptualize identity-specific stressors as additive, engendering more complicated and divisive forms of risk for mental health conditions. Essentially, individuals who experience high levels of poverty-related stress are required to cope with generalized stressors experienced by most people as well as threats and concerns stemming from environmental, financial, and psychosocial hardships associated with a lower socioeconomic identity status. To further substantiate our model of poverty-related stress as a unique platform by which theorists, researchers, and clinicians can evaluate identity-related strain, it will be important to determine whether our observed factors account for variation in depressive, anxious, substance-related, somatic, and psychotic disorders over and above the effects of generalized stress measures.

## 5. Conclusion

This first attempt to develop and validate a model and psychometrically sound measure for poverty-related stress (c.f. the Appendix) netted promising results. Our study is aimed at addressing the gap within the literature by developing an instrument that assesses domain-specific poverty-related stressors and their relationship with mental health outcomes. The results supported the notion that poverty-related stress is a function of a dynamic interaction between stressors and not just based on the mere presence of such. Moreover, results validate our poverty-related stress dimensions as risk factors for different debilitative health outcomes. Overall, our study provides interesting avenues for future research and a means to inform policy and clinical interventions.

## Figures and Tables

**Table 1:** Demographic characteristics of participants.

Item	Category	Study 1: EFA		Study 2: CFA		Study 3: validity	
		*n*	%	*n*	%	*n*	%
Sex assigned at birth	Male	96	46.6	184	46.0	108	49.3
	Female	110	53.4	215	53.9	111	50.7

Gender identity	Man	97	47.1	182	45.5	106	48.4
	Woman	107	51.9	211	52.8	111	50.7
	Genderqueer	2	1.0	7	1.8	2	0.9

Age (years)	18-20 years	3	1.5	5	1.3	2	1.4
	21-30 years	72	35.0	154	38.5	54	24.7
	31-40 years	75	36.4	135	33.8	79	36.1
	41-50 years	31	15.0	60	15.0	39	17.8
	51-60 years	18	8.7	29	7.3	29	13.2
	61+ years	7	3.4	14	3.5	15	6.8

Ethnic identity	Caucasian/White	125	60.7	263	65.8	174	79.5
	African American/Black	48	23.3	68	17.0	20	9.1
	Asian/Asian American	18	8.7	34	8.5	13	5.9
	Mexican American/Latino(a)	7	3.4	17	4.3	6	2.7
	American Indian/Native American	3	1.5	0	0.0	1	0.5
	Multiethnic	4	1.9	13	3.3	4	1.8
	Other	1	0.5	5	1.3	1	0.5

Marital status	Single	77	37.4	175	43.8	81	37.0
	Married/partnered	109	52.9	188	47.0	116	53.0
	Separated	1	0.5	3	0.8	0	0.0
	Divorced	16	7.8	28	7.0	16	7.3
	Widowed	1	0.5	5	1.3	6	2.7

Education	Less than high school	0.0	0.0	5	1.3	0.0	0.0
	Some high school	0.0	0.0	14	3.5	1	0.5
	High school diploma/GED	37	18.0	58	14.5	19	8.7
	Some college/vocational school	56	27.2	101	25.3	37	16.9
	Vocational degree	8	3.9	24	6.0	20	9.1
	College degree	72	35.0	152	38.0	114	52.1
	Master's degree	26	12.6	43	10.8	27	12.3
	Doctoral degree	1	0.5	3	0.8	1	0.5

**Table 2:** The 30-item measures: exploratory factor analysis and factor loading scores.

	Items	Stressor Type	EFA Model			
			*λ*_1_	*λ*_2_	*λ*_3_	*d*
1	*I had difficulty sleeping or doing other important things due to noise disturbances inside my home (e.g., crying infants and loud family members).*	PHY	**0.67**	0.02	0.07	0.48
2	*I had difficulty sleeping or doing other important things due to noise disturbances outside my home (e.g., loud neighbors, construction, neighborhood violence, public transportation, and car alarms).*	PHY	**0.70**	-0.10	0.15	0.49
3	*I was reluctant to go home or return home because the noise in my house was uncomfortably loud.*	PHY	**0.68**	0.21	-0.05	0.32
4	*I felt the need to get up and leave when it became noisy in my house.*	PHY	**0.76**	0.13	0.01	0.27
5	*I have felt stressed, irritable, or fatigued by the noise in my home.*	PHY	**0.70**	0.03	0.11	0.41
6	I have felt overwhelmed by the amount of people living in my home.	PSYSO	**0.38**	**0.37**	0.03	0.50
7	I was prone to sickness because of the amount of people living in my home.	PHY	**0.43**	**0.46**	-0.07	0.36
8	I had difficulty finding time to be alone because of the number of people in my home.	PHY	**0.54**	0.05	0.13	0.58
9	I have difficulty accessing the toilet, showers, laundry, or other resources due to the amount of people in my home.	PHY	**0.30**	0.**57**	-0.04	0.36
10	*I had to let go of some hopes and dreams to meet my most basic needs (shelter, food, clothing, etc.)*	PSYSO	0.13	0.06	**0.58**	0.54
11	I have been without water, heating, electricity, or other basic necessities because there was not enough money.	FIN	0.01	**0.61**	**0.33**	0.36
12	*Maintenance workers have condemned or threatened to condemn my home due to structural problems, poor maintenance, or other physical hazards associated with the building itself.*	PHY	0.12	**0.80**	-0.08	0.27
13	I have stayed in a homeless shelter, church, other public place, or another person's home because my home was not suitable to live in.	PHY	-0.15	**0.99**	-0.02	0.22
14	*My family and I have been threatened with eviction.*	PSYSO	-0.05	**0.84**	0.10	0.29
15	*I have worried about how difficult it would be to move if I had to move suddenly.*	PSYSO	0.23	-0.02	**0.55**	0.54
16	Relationships with family members end on bad terms.	PSYSO	**0.31**	**0.30**	**0.30**	0.44
17	Disagreements in my family often lead to violent actions (e.g., loud arguments and physical confrontations).	PSYSO	**0.28**	**0.44**	**0.24**	0.35
18	*I avoid people living in my home as much as possible.*	PSYSO	0.02	**0.64**	0.12	0.50
19	Two or more people in my family have chosen not to communicate with one another.	PSYSO	**0.41**	0.20	**0.27**	0.46
20	I encountered physical confrontations (i.e., fighting) in my home.	PSYSO	**0.19**	**0.53**	**0.23**	0.34
21	A family member or family friend moved away because they could not afford to stay in their home.	FIN	0.09	**0.45**	**0.31**	0.50
22	I have experienced a family member or family friend pass away before their time.	PSYSO	0.02	**0.24**	**0.52**	0.56
23	*I have not felt as close to a family member or family friend because they are in jail.*	PSYSO	0.01	**0.74**	0.10	0.37
24	Conflicts in my home make me feel disconnected from loved ones.	PSYSO	**0.28**	**0.43**	**0.22**	0.40
25	*Financial stress has negatively impacted my family's relationship.*	FIN	**0.22**	-0.03	**0.66**	0.40
26	I have been concerned with my appearance because my clothing appears torn, tattered, patched, was received second-hand, or is one or more years old.	PSYSO	-0.11	**0.49**	**0.60**	0.31
27	I have gone hungry because there was not enough food to eat.	FIN	-0.07	**0.54**	**0.47**	0.38
28	*I had to take advantage of available garbage bins, charities, soup kitchens, or free events in order to eat.*	FIN	0.13	**0.69**	0.09	0.31
29	*I have been forced to stay in a homeless shelter, church, other public place, or another person's home.*	FIN	0.01	**0.74**	0.08	0.39
30	*I had to sacrifice or make tough decisions because of a lack of money.*	FIN	0.03	0.18	**0.67**	0.40

Note: Bold = statistically significant (*p* < 0.05). Stressor types: PHY (physical), PSYSO (psychosocial), FIN (financial); Italicized = in final measure.

**Table:** (a) Study 1: descriptive statistics and Pearson correlations

No.	Factor	Valid (*N*)	Mean	Std. deviation	Variance	Skewness	Kurtosis	Shapiro-Wilk	1	2	3
1	Poverty-related stress	206	2.15	0.69	0.48	0.16	-0.96	0.97	—		
2	Noise disturbance	206	2.11	0.78	0.61	0.28	-0.84	0.95	0.88	—	
3	Home dysfunction	206	1.80	0.81	0.66	0.83	-0.52	0.87	0.87	0.71	—
4	Financial distress	206	0.83	0.83	0.69	-0.16	-0.80	0.96	0.82	0.58	0.53

Note: All correlations were significant at the .01 level. Mean scores were created and used.

**Table:** (b) Study 2: descriptive statistics and Pearson correlations

No.	Factor	Valid (*N*)	Mean	Std. deviation	Variance	Skewness	Kurtosis	Shapiro-Wilk	1	2	3
1	Poverty-related stress	400	0	0.91	0.84	0.81	-0.22	0.92	—		
2	Noise disturbance	400	0	0.95	0.90	0.58	-0.60	0.94	0.94	—	
3	Home dysfunction	400	0	0.96	0.92	1.02	-0.03	0.86	0.97	0.84	—
4	Financial distress	400	0	0.91	0.83	0.00	-0.85	0.98	0.61	0.52	0.53

Note: All correlations were significant at the .01 level. Mean scores were created and used.

**Table:** (c) Study 3: descriptive statistics and Pearson correlations

No.	Factor	Valid (*N*)	Std. dev	Var.	Skew.	Kurt.	Shapiro-Wilk	1	2	3	4	5	6	7	8	9	10	11	12	13
Time 1																				
1	Poverty-related stress	470	0.98	0.96	0.91	-0.39	0.86	--												
2	Noise disturbance	470	0.95	0.91	0.82	-0.46	0.89	0.96	--											
3	Home dysfunction	470	0.97	0.93	1.05	-0.41	0.77	0.95	0.90	--										
4	Financial distress	470	0.95	0.90	0.54	-0.87	0.91	0.88	0.84	0.81	--									
5	Depression	470	0.63	0.40	0.79	-0.59	0.87	0.91	0.85	0.81	0.83	—								
6	Anxiety	470	0.61	0.37	0.75	-0.75	0.86	0.95	0.89	0.86	0.84	0.94	—							
7	Flourishing	470	1.24	1.54	-0.95	0.85	0.94	-0.13	-0.11	-0.11	-0.20	-0.31	-0.25	—						
Time 2																				
8	Poverty-related stress	219	0.96	0.92	0.86	-0.57	0.86	0.97	0.95	0.94	0.87	0.85	0.92	-0.16	--					
9	Noise disturbance	219	1.21	1.45	0.75	-0.67	0.88	0.93	0.91	0.88	0.80	0.79	0.86	-0.18	0.95	--				
10	Home dysfunction	219	1.14	1.31	1.07	-0.13	0.80	0.95	0.91	0.93	0.80	0.80	0.87	-0.13	0.96	0.92	--			
11	Financial distress	219	1.07	1.13	0.72	-0.74	0.89	0.95	0.92	0.89	0.87	0.83	0.89	-0.17	0.96	0.91	0.91	--		
12	Depression	219	0.52	0.27	0.76	-0.69	0.87	0.85	0.78	0.75	0.76	0.95	0.91	-0.32	0.85	0.80	0.79	0.83	—	
13	Anxiety	219	0.78	0.61	0.81	-0.63	0.86	0.92	0.86	0.84	0.81	0.93	0.98	-0.21	0.93	0.88	0.88	0.90	0.95	—
14	Flourishing	219	1.14	1.30	-1.21	1.89	0.92	**-0.07**	-0.05	-0.02	-0.13	-0.32	-0.21	0.92	**-0.07**	**-0.08**	**-0.02**	-0.09	-0.35	-0.18

Bold = nonstatistically significant (*p* > 0.05). Note: factor scores were created and used.

**Table 4:** Model fit statistics.

Fit indices	Cut-off criterion	Sensitive to *N*	Penalty for model complexity
Absolute fit indices			
Chi-square (*χ*^2^)	(i) Lowest comparative value between measurement models (ii) Nonsignificant chi-square (*p* < 0.01) (iii) Significant difference in chi-square between models (iv) For model comparison: retain the model with the lowest chi-square	Yes	Yes

Approximate fit indices			
Root mean square error of approximation (RMSEA)	(i) 0.06 to 0.08 (marginally acceptable); 0.01 to 0.05 (excellent) (ii) Not-significant (*p* > 0.01) (iii) 90% confidence interval Rande should not include zero (iv) For invariance: retain model where ΔRMSEA ≤ 0.015	Yes	Yes
Standardized root mean square residual (SRMR)	(i) 0.06 to 0.08 (marginally acceptable); 0.01 to 0.05 (excellent)	Yes	No

Incremental fit indices			
Comparative fit index (CFI)	(i) 0.90 to 0.95 (marginally acceptable fit); 0.96 to 0.99 (excellent) (ii) For invariance: retain model with highest CFI value (ΔCFI > 0.01)	No	Yes
Tucker-Lewis Index (TLI)	(i) 0.90 to 0.95 (marginally acceptable fit); 0.96 to 0.99 (excellent) (ii) For invariance: retain model with highest TLI value (ΔTLI > 0.01)	No	Yes
Akaike information criterion (AIC)	(i) For model comparison: retain the model with the lowest value	No	No
Bayes information criterion (BIC)	(i) For model comparison: retain the model with the lowest value	No	No
Sample-size adjusted BIC (aBIC)	(i) For model comparison: retain the model with the lowest value	No	No

Note. Table adapted from Van Zyl & Ten Klooster [48].

**Table 5:** Exploratory factor analysis—factor loadings, item uniqueness, and reliability estimates.

Label	Item content	EFA model 1			
		*λ*_1_	*λ*_2_	*λ*_3_	
Noise disturbance					
PRSS 1	I had difficulty sleeping or doing other important things due to noise disturbances inside my home (e.g., crying infants and loud family members).	**0.66**	0.00	0.15	0.43
PRSS 2	I had difficulty sleeping or doing other important things due to noise disturbances outside my home (e.g., loud neighbors, construction, neighborhood violence, public transportation, and car alarms).	**0.68**	-0.06	0.19	0.44
PRSS 3	I was reluctant to go home or return home because the noise in my house was uncomfortably loud.	**0.74**	0.18	-0.07	0.3
PRSS 4	I felt the need to get up and leave when it became noisy in my house.	**0.73**	0.16	0.02	0.28
PRSS 5	I have felt stressed, irritable, or fatigued by the noise in my home.	**0.67**	0.04	0.16	0.4

Home dysfunction					
PRSS 6	Maintenance workers have condemned or threatened to condemn my home due to structural problems, poor maintenance, or other physical hazards associated with the building itself.	0.11	**0.85**	-0.18	0.24
PRSS 7	My family and I have been threatened with eviction.	-0.03	**0.83**	0.04	0.32
PRSS 8	I avoid people living in my home as much as possible	-0.06	**0.74**	0.02	0.5
PRSS 9	I have not felt as close to a family member or family friend because they are in jail.	-0.02	**0.78**	0.06	0.38
PRSS 10	I had to take advantage of available garbage bins, charities, soup kitchens, or free events in order to eat.	0.15	**0.72**	-0.01	0.31
PRSS 11	I have been forced to stay in a homeless shelter, church, other public place, or another person's home.	0.02	**0.74**	0.06	0.38

Financial distress					
PRSS 12	I had to let go of some hopes and dreams to meet my most basic needs (shelter, food, clothing, etc.)	0.17	-0.01	**0.7**	0.49
PRSS 13	I have worried about how difficult it would be to move if I had to move suddenly.	0.03	0.16	**0.6**	0.5
PRSS 14	Financial stress has negatively impacted my family's relationship.	0.18	0.02	**0.6**	0.38
PRSS 15	I had to sacrifice or make tough decisions because of a lack of money.	-0.01	0.25	**0.65**	0.41

Eigenvalues, variances, and reliability estimates					
	Eigenvalues	7.75	1.57	1.10	
	*R* ^2^ (%)	51.69	10.46	7.27	
	McDonald's omega	0.89	0.91	0.83	
	Cronbach's alpha	0.88	0.91	0.83	

Bolded factor loading scores indicate a retained item with the associated factor score.

**Table 6:** Competing measurement models.

Model type	*χ* ^2^	df	CFI	TLI	RMSEA [Est.; CI]	SRMR	AIC	BIC	aBIC	Meets model fit criteria	Meets measure quality criteria
(1) Unidimensional model	678.70	90	0.81	0.78	0.13; [0.12-0.14]	0.09	14015.08	14194.70	14051.91	No	No
(2) Three correlated first-order factor model	197.79	87	0.96	0.96	**0.06**; [0.05-0.07]	0.04	13540.17	13731.76	13579.45	Yes	Yes
(3) Second-order model with three first-order factors	197.79	87	0.96	0.96	**0.06**; [0.05-0.07]	0.04	13540.17	13731.76	13579.45	Yes	Yes
(4) Biactor model (one general & three specific factors	153.12	75	0.98	0.96	**0.05**; [0.04-0.06]	0.04	13519.50	13758.99	13568.60	Yes	No
(5) ESEM model with three first-order factors	**86.05**	63	0.99	0.99	**0.03**; [0.01-0.05]	0.02	13476.43	13763.82	13535.36	Yes	Yes
(6) H-ESEM model with three first-order factors	**86.05**	63	0.99	0.99	**0.03**; [0.01-0.05**]**	0.02	13476.43	13763.82	13535.36	Yes	Yes
(7) Bifactor ESEM model (one general & three specific factors)	**52.30**	51	1.00	1.00	**0.01**; [< 0.01-0.03]	0.01	13466.68	13801.96	13535.42	Yes	No

Note: *χ*^2^ = chi-square; df = degrees of freedom; TLI = Tucker-Lewis Index; CFI = comparative fit index; RMSEA = root mean square error of approximation [90% CI]; SRMR = standardized root mean square residual; AIC = Akaike information criterion; BIC = Bayes information criterion; aBIC = adjusted Bayes information criterion. Bold: nonsignificant *p* > 0.01. For convergence, first-order factor loadings were constrained to be equal.

**Table 7:** Standardized factor loadings and parameter estimates.

Item label	H-ESEM model 6								
	Noise disturbance			Home dysfunction			Financial distress		
	*λ*_1_	S.E	*p*	*λ*_2_	S.E	*p*	*λ*_3_	S.E	*p*
PRSS1	**0.59**	0.08	0.001	0.05	0.09	0.573	0.07	0.06	0.209
PRSS2	**0.51**	0.09	0.001	0.03	0.09	0.770	0.22	0.06	0.001
PRSS3	**0.75**	0.07	0.001	0.15	0.08	0.053	-0.06	0.05	0.200
PRSS4	**0.84**	0.02	0.001	0.07	0.00	0.001	-0.11	0.01	0.001
PRSS5	**0.77**	0.08	0.001	-0.07	0.09	0.449	0.12	0.06	0.032
PRSS6	0.20	0.08	0.007	**0.69**	0.07	0.001	-0.15	0.05	0.002
PRSS7	0.14	0.07	0.051	**0.67**	0.07	0.001	0.03	0.05	0.507
PRSS8	0.15	0.09	0.080	**0.36**	0.09	0.001	0.15	0.06	0.009
PRSS9	0.07	0.08	0.406	**0.68**	0.07	0.001	-0.01	0.05	0.782
PRSS10	-0.11	0.09	0.205	**0.80**	0.08	0.001	0.09	0.05	0.062
PRSS11	-0.20	0.01	0.001	**0.99**	0.02	0.001	0.00	0.00	0.001
PRSS12	0.07	0.09	0.467	0.02	0.09	0.833	**0.69**	0.05	0.001
PRSS13	0.11	0.10	0.256	-0.02	0.10	0.841	**0.65**	0.05	0.001
PRSS14	0.10	0.09	0.303	0.01	0.09	0.911	**0.68**	0.05	0.001
PRSS15	-0.15	0.01	0.001	0.09	0.01	0.001	**0.76**	0.03	0.001
Poverty-related stress factor loadings	0.86	0.06	0.001	0.90	0.07	0.001	0.52	0.06	0.001
Variances & reliability estimates									
*R*^2^ (%)	0.74			0.81			0.27		
McDonald's omega	0.87			0.88			0.81		
Cronbach's alpha	0.86			0.88			0.81		

Bolded factor loading scores indicate a retained item with the associated factor.

**Table 8:** Longitudinal confirmatory factor analysis: measurement model fit statistics for time 1 and time 2.

Model type	*χ* ^2^	df	CFI	TLI	RMSEA [Est.; CI[	SRMR	AIC	BIC	aBIC	Meets model fit criteria	Meets measure quality criteria
(1) LFA unidimensional model	1920.49	404	0.83	0.82	0.09; [0.085-0.093]	0.07	19366.06	19743.96	19455.14	No	No
(2) LFA three correlated first-order factor model	1366.81	396	0.89	0.88	0.07; [0.068-0.076]	0.40	18828.37	19239.49	18925.29	No	Yes
(3) LFA second-order model with three first-order factors	1298.92	398	0.90	0.89	0.07; [0.065-0.074]	0.06	18756.48	19159.30	18851.44	No	Yes
(4) LFA bifactor model (one general & three specific factors	942.39	365	0.94	0.92	0.06; [0.053-0.063]	0.04	18465.96	19005.81	18593.22	Yes	No
(5) LFA ESEM model with three first-order factors	1137.79	348	0.91	0.90	0.07; [0.065-0.074]	0.37	18695.36	19305.81	18839.26	Yes	Yes
(6) LFA H-ESEM model with three first-order factors	1102.72	350	0.92	0.90	0.07; [0.063-0.072]	0.06	18656.29	19258.44	18798.24	Yes	Yes
(7) LFA bifactor ESEM model (one general & three specific factors)	—	—	—	—	—	—	—	—	—	—	—

Note. The LFA bifactor ESEM model could not converge. As a result, no data are furnished in the current table.

**(a):** 

Model type	*χ* ^2^	df	CFI	TLI	RMSEA [Est.; CI]	SRMR	Meets fit criteria	Meets invariance criteria
M1-configural invariance	889.28	342	0.94	0.92	0.06; [0.054-0.063]	0.07	Yes	Yes
M2-metric invariance	1016.05	375	0.93	0.92	0.06; [0.056-0.065]	0.40	Yes	Yes
M3-scalar invariance	1047.53	387	0.93	0.92	0.06; [0.056-0.065]	0.06	Yes	Yes

**(b):** 

Model comparisons					
Comparison	Δ*χ*^2^	ΔCFI	ΔTLI	ΔRMSEA	ΔSRMR
M3 vs. M1	158.25	-0.01	-0.01	<0.01	0.01
M2 vs. M1	126.77	-0.01	-0.01	0.01	0.01
M3 vs. M2	31.48	<0.01	<0.01	<0.01	<0.01

Note. Models were compared based on Chen's [73] criteria: changes in RMSEA (Δ < 0.015; *p* > 0.01), SRMR (Δ < 0.02 for configural versus metric/scalar; Δ < 0.01 for metric versus scalar), CFI (Δ < 0.01), and TLI (Δ < 0.01).

**Table 10:** Concurrent and predictive validity: the relationships between poverty-related stress and theoretically salient concepts (depression, anxiety, and flourishing).

Regression path			Standardized			*R* ^2^	Validity established
			*β*	S.E.	*t*		
Concurrent validity							
Poverty-related stress time 1	➔	Depression time 1	0.85⁣^∗∗^	0.02	48.12	0.71	Yes
Poverty-related stress time 1	➔	Anxiety time 1	0.94⁣^∗∗^	0.01	81.83	0.88	Yes
Poverty-related stress time 1	➔	Flourishing time 1	-0.15⁣^∗∗^	0.04	-3.44	0.02	Yes
Poverty-related stress time 2	➔	Depression time 2	0.68⁣^∗∗^	0.04	16.43	0.46	Yes
Poverty-related stress time 2	➔	Anxiety time 2	0.91⁣^∗∗^	0.02	47.08	0.83	Yes
Poverty-related stress time 2	➔	Flourishing time 2	-0.21⁣^∗∗^	0.06	-3.47	0.04	Yes
Predictive validity							
Poverty-related stress time 1	➔	Depression time 2	0.58⁣^∗∗^	0.05	11.41	0.33	Yes
Poverty-related stress time 1	➔	Anxiety time 2	0.74⁣^∗∗^	0.03	28.29	0.55	Yes
Poverty-related stress time 1	➔	Flourishing time 2	-0.24⁣^∗∗^	0.06	-4.29	0.06	Yes

Note: *β* = standardized beta; S.E. = standard error; *R*^2^ = variance, ⁣^∗∗^ = *p* is significant at the 0.01 level.

**Table 11:** Concurrent and predictive validity: the relationships between poverty-related stress dimensions and theoretically salient concepts (depression, anxiety, and flourishing).

Regression path			Standardized			*R* ^2^
			*β*	S.E.	*t*-value	
Concurrent validity						
Noise disturbance time 1	➔	Depression time 1	0.22⁣^∗∗^	0.16	1.35	0.68
Home dysfunction time 1	➔	Depression time 1	0.11⁣^∗∗^	0.15	0.72	
Financial distress time 1	➔	Depression time 1	0.55⁣^∗∗^	0.14	3.96	
Noise disturbance time 1	➔	Anxiety time 1	0.19⁣^∗∗^	0.18	1.07	0.80
Home dysfunction time 1	➔	Anxiety time 1	0.37⁣^∗∗^	0.17	2.24	
Financial distress time 1	➔	Anxiety time 1	0.41⁣^∗∗^	0.12	3.45	
Noise disturbance time 1	➔	Flourishing time 1	-0.32⁣^∗∗^	0.21	-1.52	0.08
Home dysfunction time 1	➔	Flourishing time 1	-0.07	0.13	-0.49	
Financial distress time 1	➔	Flourishing time 1	-0.44⁣^∗∗^	0.17	-2.61	
Noise disturbance time 2	➔	Depression time 2	0.46⁣^∗∗^	0.15	2.87	0.46
Home dysfunction time 2	➔	Depression time 2	0.16⁣^∗∗^	0.11	1.64	
Financial distress time 2	➔	Depression time 2	0.42⁣^∗∗^	0.10	3.51	
Noise disturbance time 2	➔	Anxiety time 2	0.42⁣^∗∗^	0.15	2.87	0.72
Home dysfunction time 2	➔	Anxiety time 2	0.18⁣^∗∗^	0.11	1.64	
Financial distress time 2	➔	Anxiety time 2	0.35⁣^∗∗^	0.10	3.51	
Noise disturbance time 2	➔	Flourishing time 2	-0.47⁣^∗∗^	0.20	-2.31	0.13
Home dysfunction time 2	➔	Flourishing time 2	-0.43⁣^∗∗^	0.20	-2.12	
Financial distress time 2	➔	Flourishing time 2	-0.18⁣^∗∗^	0.11	-1.67	
Predictive validity						
Noise disturbance time 1	➔	Depression time 2	0.15	0.23	0.66	0.34
Home dysfunction time 1	➔	Depression time 2	0.02	0.22	0.08	
Financial distress time 1	➔	Depression time 2	0.45⁣^∗∗^	0.13	0.13	
Noise disturbance time 1	➔	Anxiety time 2	0.25⁣^∗∗^	0.21	1.19	0.55
Home dysfunction time 1	➔	Anxiety time 2	0.68⁣^∗∗^	0.24	2.85	
Financial distress time 1	➔	Anxiety time 2	0.36⁣^∗∗^	0.22	1.59	
Noise disturbance time 1	➔	Flourishing time 2	-0.54⁣^∗∗^	0.29	-1.82	0.16
Home dysfunction time 1	➔	Flourishing time 2	-0.60⁣^∗∗^	0.32	-1.87	
Financial distress time 1	➔	Flourishing time 2	-0.32⁣^∗∗^	0.22	-1.47	

Note: *β* = standardized beta; S.E. = standard error; *R^2^* = variance, ⁣^∗∗^ = *p* is significant at the 0.01 level.

**Table 12:** The final iteration of the Poverty-Related Stress Scale.

Item #	Item	Never	Sometimes	Often	Always
Noise disturbance items					
PRSS 1	I had difficulty sleeping or doing other important things due to noise disturbances inside my home (e.g., crying infants and loud family members).	1	2	3	4
PRSS 2	I had difficulty sleeping or doing other important things due to noise disturbances outside my home (e.g., loud neighbors, construction, neighborhood violence, public transportation, and car alarms).	1	2	3	4
PRSS 3	I was reluctant to go home or return home because the noise in my house was uncomfortably loud.	1	2	3	4
PRSS 4	I felt the need to get up and leave when it became noisy in my house.	1	2	3	4
PRSS 5	I have felt stressed, irritable, or fatigued by the noise in my home.	1	2	3	4

Home dysfunction items					
PRSS 6	Maintenance workers have condemned or threatened to condemn my home due to structural problems, poor maintenance, or other physical hazards associated with the building itself.	1	2	3	4
PRSS 7	My family and I have been threatened with eviction.	1	2	3	4
PRSS 8	I avoid people living in my home as much as possible.	1	2	3	4
PRSS 9	I have not felt as close to a family member or family friend because they are in jail.	1	2	3	4
PRSS 10	I had to take advantage of available garbage bins, charities, soup kitchens, or free events in order to eat.	1	2	3	4
PRSS 11	I have been forced to stay in a homeless shelter, church, other public place, or another person's home.	1	2	3	4

Financial distress items					
PRSS 12	I had to let go of some hopes and dreams to meet my most basic needs (shelter, food, clothing, etc.)	1	2	3	4
PRSS 13	I have worried about how difficult it would be to move if I had to move suddenly.	1	2	3	4
PRSS 14	Financial stress has negatively impacted my family's relationship.	1	2	3	4
PRSS 15	I had to sacrifice or make tough decisions because of a lack of money.	1	2	3	4

## Data Availability

Data will be made available upon request by contacting the corresponding researcher.

## Appendix

The PRSS is available for free to researchers, educators, and service providers for noncommercial purposes, such as education and research. We kindly request that users share their data, including raw test scores for the PRSS and relevant sociodemographic information of the surveyed samples, to aid in the ongoing validation of the PRSS's psychometric properties.

Instructions: Please rate the extent to which you experienced these stressors in the last five (5) years. Choose the descriptor that best corresponds with your lived experience (Table 12).
